# Performance of generative artificial intelligence in oral and maxillofacial radiology based on the board-certification examination of Japan

**DOI:** 10.1007/s11282-026-00965-z

**Published:** 2026-08-20

**Authors:** Yohei Takeshita, Toshiyuki Kawazu, Miki Hisatomi, Mamiko Fujikura, Shunsuke Okada, Akira Shibanuma, Yuri Namba, Suzuka Yoshida, Saori Yoshida, Yoshihide Nakamura, Yoshinobu Yanagi

**Affiliations:** 1 Department of Oral and Maxillofacial Radiology, Faculty of Medicine, Dentistry and Pharmaceutical Sciences, Okayama University, 2-5-1 Shikata-Cho, Kita-Ku, Okayama, 700-8558 Japan; 2 Department of Oral and Maxillofacial Radiology, Medical Development Field, Okayama University, 2-5-1 Shikata-Cho, Kita-Ku, Okayama, 700-8558 Japan; 3 Department of Community and Global Health, Graduate School of Medicine, The University of Tokyo, 7-3-1 Hongo, Bunkyo-Ku, Tokyo, 113-0033 Japan; 4 Department of Oral and Maxillofacial Radiology, Division of Dentistry, Okayama University Hospital, 2-5-1 Shikata-Cho, Kita-Ku, Okayama, 700-8558 Japan; 5 Preliminary Examination Room, Medical Development Field, Okayama University, 2-5-1 Shikata-Cho, Kita-Ku, Okayama, 700-8558 Japan; 6 Department of Oral and Maxillofacial Radiology, Graduate School of Medicine, Dentistry and Pharmaceutical Sciences, Okayama University, 2-5-1 Shikata-Cho, Kita-Ku, Okayama, 700-8558 Japan

**Keywords:** Artificial intelligence, Generative artificial intelligence, ChatGPT, Large language model, Radiology, Education

## Abstract

**Objective:**

To evaluate the performance and potential utility of generative artificial intelligence (AI) in oral and maxillofacial radiology using the board-certification examination administered by the Japanese Society for Oral and Maxillofacial Radiology (JSOMR).

**Methods:**

The responses generated by ChatGPT for multiple-choice questions from the board-certification examination of the JSOMR over the three-year period from 2020 to 2022 were assessed. The questions were manually entered individually as prompts for GPT-3.5, GPT-4, and GPT-5, which are the models available from ChatGPT. The accuracy was calculated according to examination year, question format, and level of taxonomy.

**Results:**

GPT-3.5 achieved an accuracy of 40.3% for the three years (42.9%, 42.0%, and 36.0% for 2020, 2021, and 2022, respectively), that of GPT-4 was 67.8% (67.3%, 74.0%, and 62.0%, respectively), and that of GPT-5 was 76.5% (79.6%, 78.0%, and 72.0%, respectively). GPT-5’s results exceeded the passing score for each year with the accuracy significantly outperformed that of GPT-3.5 and GPT-4. Regarding performance according to the question format, GPT-5 performed significantly superior to the earlier models, especially on two-answer questions.

**Conclusions:**

On the board-certification examination of the JSOMR, the performance of GPT-5 was significantly superior to that of GPT-3.5 and GPT-4. This suggests that, given the rapid development of generative AI, GPT-5 has reached a level of text-based knowledge equivalent to that assessed in the board-certification examination of the JSOMR.

## Introduction

Generative artificial intelligence (AI) has recently attracted a great deal of attention. It is exemplified by ChatGPT developed by OpenAI (San Francisco, CA, USA), which is a tool based on a large language model (LLM) [1]. Generative AI is now increasingly utilized not only for digital content production and advertising, but also in the fields such as medical and healthcare [2–5]. An LLM is a form of AI designed for natural language processing, although its ability to process data beyond text including images and audio is limited because it was developed for generating text data [3, 6–8]. The generative pre-trained transformer (GPT) and related models are possible to generate human-like answers or response to user prompts based on training with extensive text data. In addition to ChatGPT, AI chatbots such as Bing, Gemini, and Claude have quickly become popular and are frequently used in daily life [5].

As of February 2026, GPT-5, which is the latest ChatGPT model, is currently available on the OpenAI website [9, 10]. Several of its characteristics differ from those of the previous GPT-3.5 and GPT-4 models. The number of parameters for GPT-3.5, GPT- 4, and GPT-5 have not been disclosed, but GPT-5 is estimated to have much larger parameters than GPT-3.5 and GPT-4. Furthermore, the maximum numbers of tokens, which is associated with the length of responses is different among models. As the number of tokens increases, ChatGPT is able to generate more extensive responses, and GPT-5 is recognized to have much larger number of tokens than those of GPT-3.5 and GPT-4.

In the field of radiology, AI techniques such as machine learning or deep learning have been primarily evaluated in the diagnostic imaging to improve the diagnostic accuracy or reduce the burden on radiologists [11]. In dentistry, it is used for tooth identification, periodontal diseases, caries detection, and implants [12]. In areas of radiology, generative AI tools including ChatGPT have been evaluated and applied not only for diagnostic imaging but also for decision making and education [13–19]. The number of studies evaluating the performance of generative AI has grown since 2023 [4, 20–22]. ChatGPT performance in medical radiology has already been evaluated using the board-style examinations in the USA and Canada as well as the board examination in Japan [6–8]. The previous work [23, 24] investigating the board-qualification examination of the Japanese Society for Oral and Maxillofacial Radiology (JSOMR), was the first to assess the ChatGPT performance using an official board examination for oral and maxillofacial radiology. The authors reported that GPT-4 achieved significantly greater accuracy than GPT-3.5 as of 2024, GPT-4 could be an effective resource for examination preparation and educational support.

The oral radiologists who want to get a status of board-certified specialist of the JSOMR and have a license of board-qualified specialist must pass the board-certification examination of the JSOMR, which including a written section composed of multiple-choice questions covering topics such as radiation oncology and diagnostic imaging, as well as an image reading section. In the written section, it contains text-only questions, and they are disclosed after the examination. Learning and preparing for this examination are challenging for examinees because the questions are much more difficult than those of the board-qualification examination and, although the passing score is disclosed as 70% by JSOMR, the correct answers are not. However, the performance of the latest GPT-5 model in oral and maxillofacial radiology examinations has not been investigated. Furthermore, it remains unclear whether improvements in GPT models translate into improved performance on specialized radiological knowledge questions and whether these models can serve as useful tools for diagnosis and education. Therefore, this study aimed to evaluate the performance of GPT-5 compared with previous GPT models using the written section of the JSOMR board-certification examination and to assess its potential utility for diagnosis and education.

## Materials and methods

ChatGPT’s responses on the written section of the JSOMR board-certification examination for the three years from 2020 to 2022 were assessed in this study. Each examination included 50 Japanese-language multiple-choice questions and contained text-based questions. A total of 150 questions were hence included.

Each question consisted of five choices, and the examinees were required to choose the correct answer (or answers) according to the instructions provided in the question. The questions included instructions specifying the number of answers to be chosen (e.g. “Choose one correct answer”, “Choose two correct answers”, “Choose three correct answers”, or “Choose all correct answers”). Accordingly, the questions were classified into four types: type X (requiring one correct answer), type X2 (requiring two correct answers), type X3 (requiring three correct answers), and type XX (requiring to choose all correct answers).

We also classified the questions according to their cognitive levels. Based on Bloom’s taxonomy and previous studies regarding the assessment of medical knowledge [24–26], the questions were categorized into three types: type I (requiring only the recall of knowledge), type II (requiring the interpretation of given information), and type III (requiring both the interpretation of the provided information and the solution of a specific problem). Only type I and type II questions were included in the analysis, as no type III questions were identified.

Because the answers were not disclosed, four board-certified specialists from the JSOMR independently reviewed the questions to determine the correct answers, consulting textbooks, scientific literature, and relevant online resources as needed. After the independent review, discrepancies among reviewers were discussed until the consensus was reached. One question was excluded from the analysis because consensus could not be reached. Therefore, the performance of ChatGPT was assessed on 149 questions.

The Japanese-language multiple-choice questions including instructions specifying the number of answers to be chosen were each manually entered into GPT-3.5, GPT-4, and GPT-5 models without any special prompts. The assessments of GPT-3.5 and GPT-4 were performed in October 2023, and the assessment of GPT-5 was performed in September 2025. Based on the answers determined by the board-certified specialists, the responses were classified as either correct or not. The performance of each GPT model was subsequently assessed with respect to examination year, question format, and level of taxonomy.

The performance of GPT-3.5, GPT-4, and GPT-5 according to year, question format, and level of taxonomy was calculated as the percentage of questions that each GPT model answered correctly. Significant differences among the models were identified using Cochran’s Q test, and pairwise comparisons were made using the McNemar test with the Holm method for multiple comparisons. These tests were calculated using R (version 4.5.1, R Foundation for Statistical Computing, Vienna, Austria), with statistical significance defined as *p* < 0.05.

## Results

### Overall accuracy of GPT-3.5, GPT-4, and GPT-5, and performance according to the examination year

**Fig. 1:**
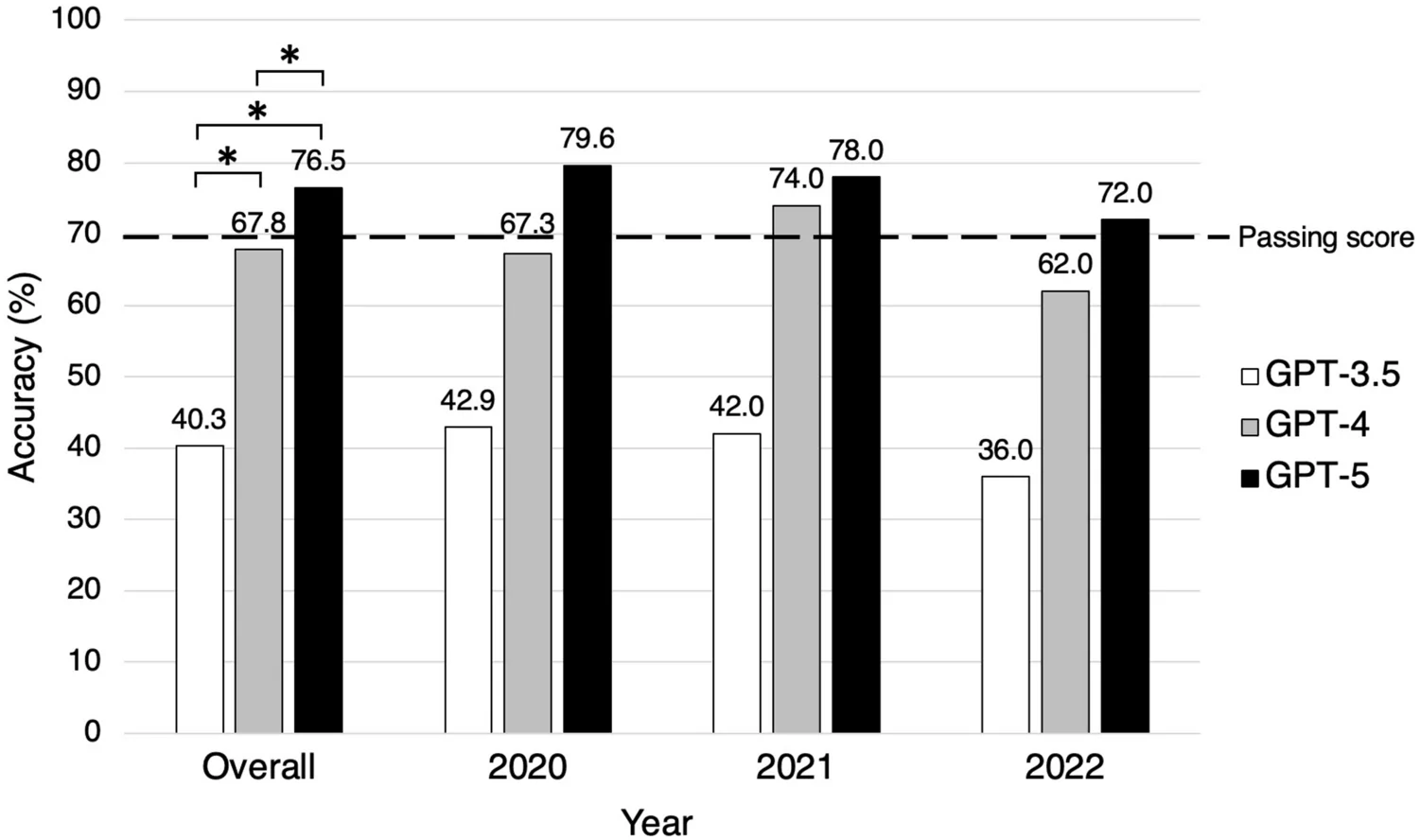
Performance comparison of three GPT models (overall and by year). GPT-5 showed significantly higher overall accuracy than that of GPT-3.5 and GPT-4 (GPT-3.5 vs GPT-4: *p* < 0.001, GPT-3.5 vs GPT-5, *p* < 0.001, GPT-4 vs GPT-5: *p* = 0.049). GPT-5 exceeded the passing score for all years

The overall accuracy for all three examination years of GPT-3.5 was 40.3% (60 of 149), that of GPT-4 was 67.8% (101 of 149), and that of GPT-5 was 76.5% (114 of 149). Significant differences were observed among the results of all models (*p* < 0.001, Cochran’s Q test); the accuracy of GPT-5 across all examination years was significantly higher than that of GPT-3.5 and GPT-4 (GPT-3.5 vs GPT-4: *p* < 0.001, GPT-3.5 vs GPT-5: *p* < 0.001, GPT-4 vs GPT-5: *p* = 0.049; McNemar test with the Holm method) (Fig. 1).

Regarding the performance for each year, GPT-3.5 achieved an accuracy for three years (from 2020 to 2022) of 42.9% (21 of 49), 42.0% (21 of 50), and 36.0% (18 of 50), respectively. Those of GPT-4 were 67.3% (33 of 49), 74.0% (37 of 50), and 62.0% (31 of 50), respectively, and those of GPT-5 were 79.6% (39 of 49), 78.0% (39 of 50), and 72.0% (36 of 50), respectively.

### Performance according to the question format

**Fig. 2:**
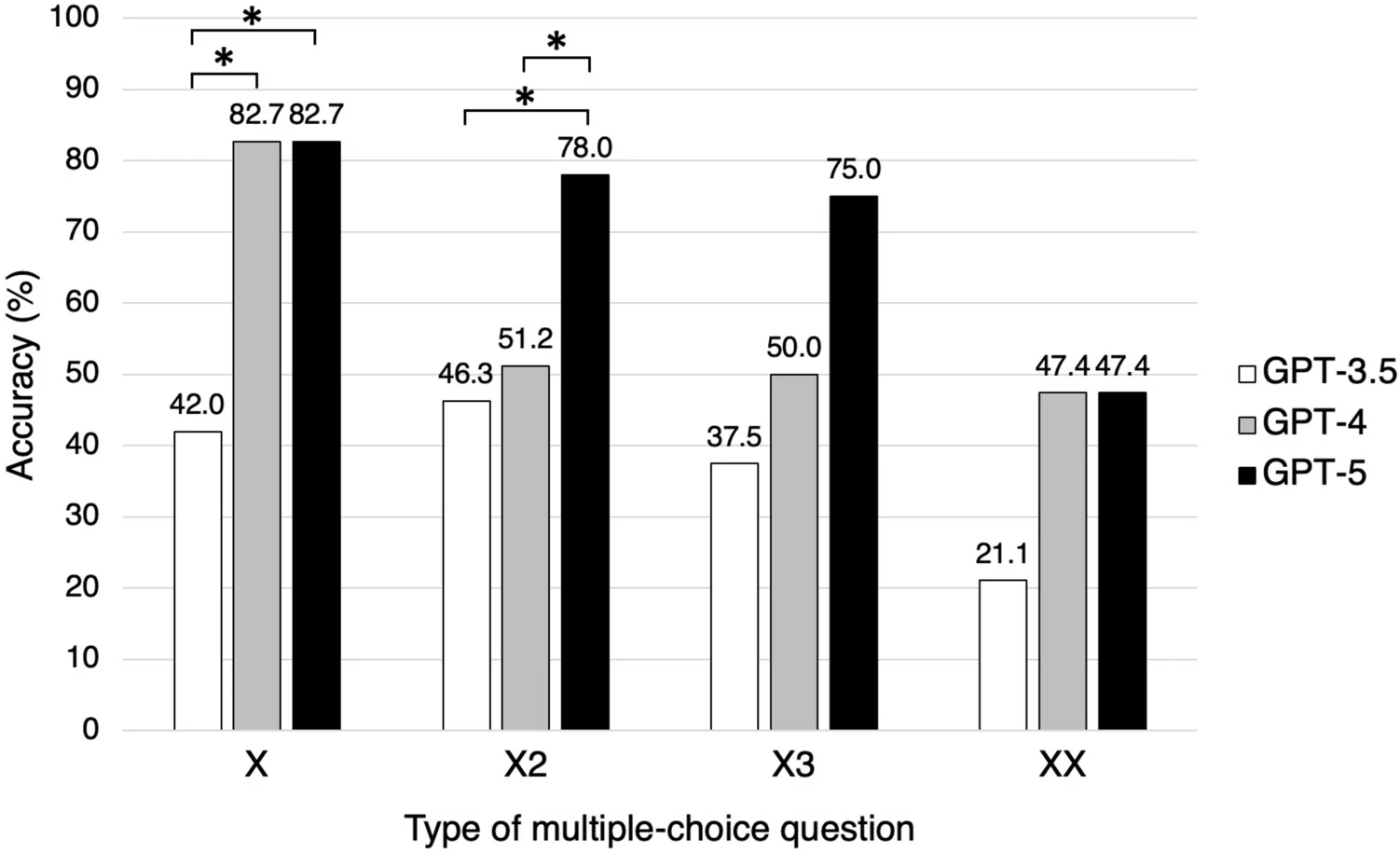
Performance comparison of three GPT models by question format. For each type of question, GPT-5 showed higher accuracy than that of GPT-3.5, and higher than or equal to that of GPT-4. For type X questions, GPT-4 and GPT-5 showed significantly higher accuracy than that of GPT-3.5 (GPT-3.5 vs GPT-4: *p* < 0.001, GPT-3.5 vs GPT-5: *p* < 0.001, GPT-4 vs GPT-5: *p* = 1). While for type X2 questions, GPT-5 showed significantly higher accuracy than that of GPT-3.5 and GPT-4 (GPT-3.5 vs GPT-4: *p* = 0.77, GPT-3.5 vs GPT-5: *p* = 0.01, GPT-4 vs GPT-5: *p* = 0.02)

The dataset for this study was including 81 type X questions, 41 type X2 questions, 8 type X3 questions, and 19 type XX questions. For each of these question types, GPT-3.5 achieved an accuracy of 42.0% (34 of 81), 46.3% (19 of 41), 37.5% (3 of 8), and 21.1% (4 of 19), respectively. The accuracy of GPT-4 for each of these question types was 82.7% (67 of 81), 51.2% (21 of 41), 50.0% (4 of 8), and 47.4% (9 of 19), respectively, and the accuracy of GPT-5 for each of these question types was 82.7% (67 of 81), 78.0% (32 of 41), 75.0% (6 of 8), and 47.4% (9 of 19), respectively. The accuracy of GPT-5 for each type of question was higher than that of GPT-3.5 and higher than or equal to that of GPT-4. Significant differences were observed among the three models for the type X and type X2 questions, whereas no significant difference was observed for type X3 and type XX questions (type X: *p* < 0.001, type X2: *p* = 0.001, type X3: *p* = 0.247, type XX: *p* = 0.103; Cochran’s Q test). GPT-4 and GPT-5 achieved significantly higher accuracy than those of GPT-3.5 on type X questions (GPT-3.5 vs GPT-4: *p* < 0.001, GPT-3.5 vs GPT-5: *p* < 0.001, GPT-4 vs GPT-5: *p* = 1; McNemar test with the Holm method), whereas GPT-5 achieved significantly higher accuracy than those of GPT-3.5 and GPT-4 on type X2 questions (GPT-3.5 vs GPT-4: *p* = 0.77, GPT-3.5 vs GPT-5: *p* = 0.01, GPT-4 vs GPT-5: *p* = 0.02; McNemar test with the Holm method) (Fig. 2).

### Performance according to the level of taxonomy

**Fig. 3:**
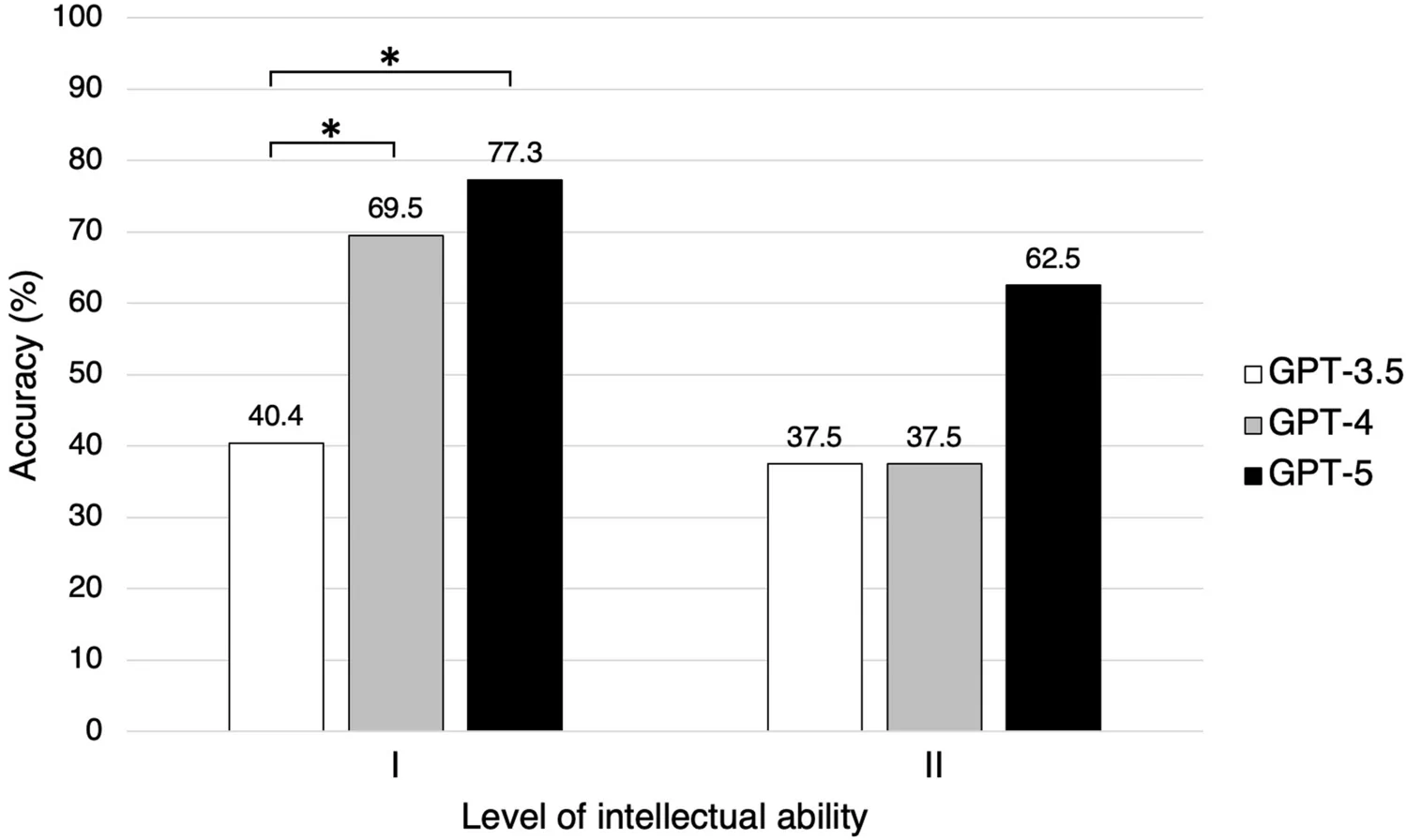
Performance comparison of three GPT models by level of taxonomy. For type I questions, GPT-5 and GPT-4 showed significantly higher accuracy than that of GPT-3.5 (GPT-3.5 vs GPT-4: *p* < 0.001, GPT-3.5 vs GPT-5: *p* < 0.001, GPT-4 vs GPT-5: *p* = 0.09)

The dataset for this study was including 141 type I questions, 8 type II questions, and there was no type III question. For type I questions requiring recall, GPT-3.5 achieved an accuracy of 40.4% (57 of 141), that of GPT-4 was 69.5% (98 of 141), and that of GPT-5 was 77.3% (109 of 141). For type II questions requiring interpretation, GPT-3.5 achieved an accuracy of 37.5% (3 of 8), that of GPT-4 was 37.5% (3 of 8), and that of GPT-5 was 62.5% (5 of 8). There were significant differences among the models for type I questions but not for type II questions (type I: *p* < 0.001, type II: *p* = 0.135; Cochran’s Q test). For type I questions, GPT-5 and GPT-4 showed significantly higher performance than that of GPT-3.5 (GPT-3.5 vs GPT-4: *p* < 0.001, GPT-3.5 vs GPT-5: *p* < 0.001, GPT-4 vs GPT-5: *p* = 0.09; McNemar test with the Holm method) (Fig. 3).

### Contents of the responses

**Fig. 4:**
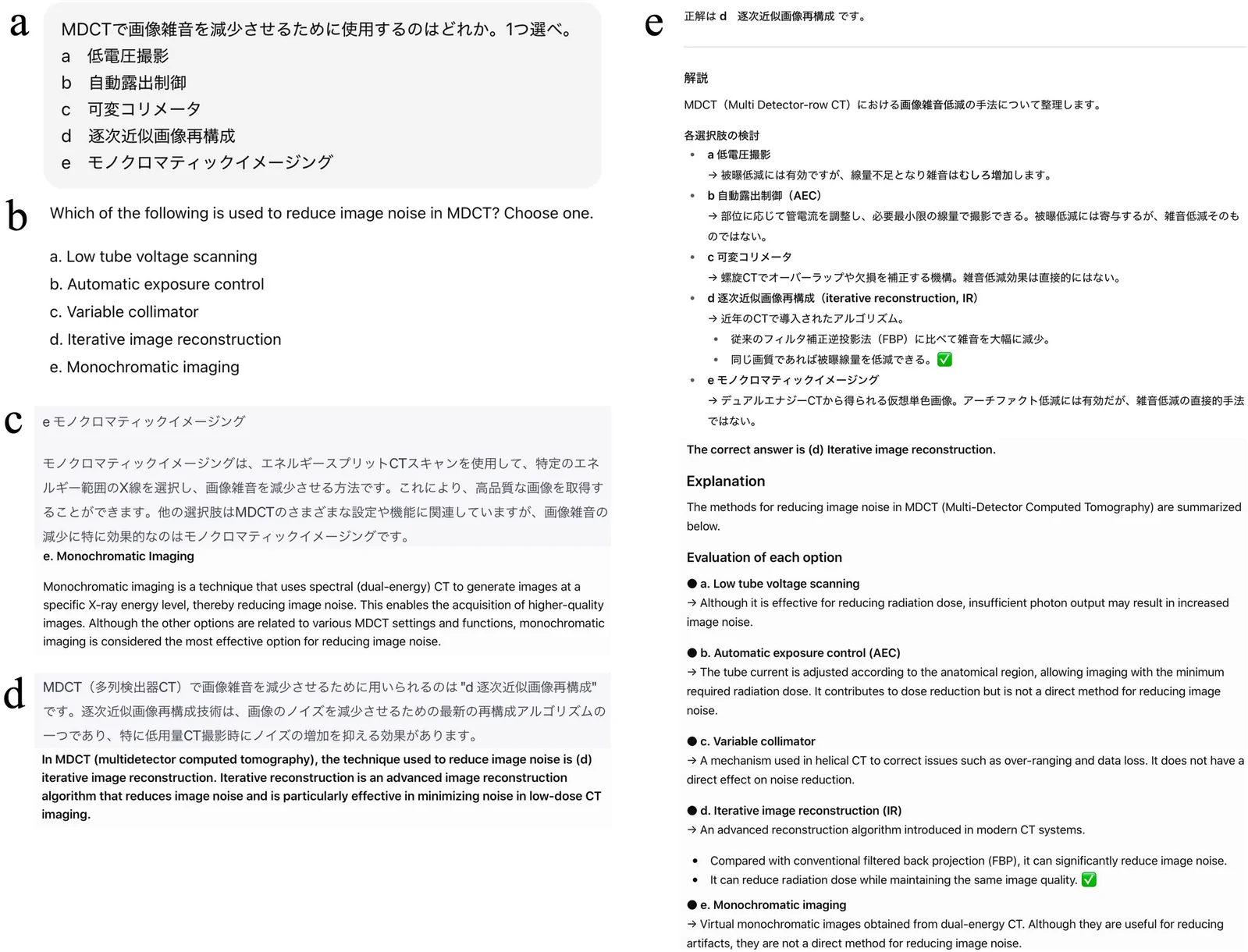
Example of the question as a prompt and the responses generated by three GPT models. **a** Japanese-language multiple-choice question was manually entered into the models. **b** English version of the question translated by ChatGPT from the original Japanese version. **c** GPT-3.5 provided the answer incorrectly with an incorrect explanation. This phenomenon is known as a hallucination. **d** GPT-4 correctly answered the question and provided a relatively detailed explanation. **e** GPT-5 correctly answered the question and provided an extremely detailed explanation that included other choices

GPT-3.5 showed a tendency to answer with incorrect responses and explanations, which is a phenomenon called “hallucination.” By contrast, GPT-4 and GPT-5 tended to provide correct detailed explanations along with the responses (Fig. 4).

## Discussion

Generative AI is now rapidly being applied in various fields including medical care, and is expected to dramatically improve accuracy and efficiency [2, 3, 13–19, 27, 28]. It has also increased in importance in the field of dentistry, and significant benefits are expected from reducing the time needed for diagnosis, treatment, and management operations. In particular, the potential of generative AI in oral and maxillofacial radiology is anticipated because it performs a key role diagnosis, which leads to correct treatment. However, information obtained from a generative AI is not guaranteed to be accurate and reliable. Generative AI is also known as a “black box” model, and hence it is more practical to evaluate its utility.

In the field of medical radiology, the effective performance of ChatGPT has previously been evaluated using the board examinations of the USA, Canada, and Japan [6–8], as well as for oral and maxillofacial radiology in Japan [24]. Oral radiologists who would like to be JSOMR board-certified specialists in Japan must pass the board-qualification examination and are then need to pass both written and image reading examination [23]. Preparing for the written examination is often challenging because the JSOMR does not release the official correct answers. We assessed the performance of GPT models, including the latest GPT-5 model, their applicability for diagnosis, and their potential for education using the written section of the JSOMR board-certification examination, which includes a wide range of radiology topics.

GPT-5 significantly outperformed GPT-3.5 and GPT-4. Although the correct answers were not disclosed, a score of 70% was disclosed to be a passing score. Hence, GPT-3.5 failed to achieve to the passing score for all three years, some of GPT-4’s scores were passing scores depending on the year, and those of GPT-5 exceeded the passing score for all years. GPT-5 had a significantly higher accuracy than GPT-3.5 and GPT-4, this result showing that this latest model achieved a performance that was the same or higher than that of JSOMR board-certified specialists, making it potentially useful for preparing and studying for the examination. There were some studies assessed the ChatGPT performance mimicking the medical radiology board examinations with four-choice questions of the USA and Canada, GPT-3.5 achieved the accuracy of 69.3% and that of GPT-4 was 80.7% [6, 7]. These scores were higher than those achieved in. Given that the JSOMR board-certification examination is for board-qualified specialists who are experienced in radiology, including clinical imaging diagnosis research and education, our results seem quite acceptable because the JSOMR board-certification examination consists of multiple-choice questions with five choices, where the number of correct answers can differ. Toyama et al. [8] assessed the performance of GPT-3.5 and GPT-4 models on medical radiology board examinations in Japan, achieving an accuracy of 40.8% for GPT-3.5 and 65.0% for GPT-4. These scores are extremely similar to those of our study, and we believe that the format of the JSOMR was appropriate for this task, even though the examination contained various types of multiple-choice questions. As found in our results, it is possible that GPT-5 could exceed the accuracy of GPT-3.5 and GPT-4 on medical radiology board examinations in Japan.

Regarding performance with respect to the question format, GPT-5 achieved higher accuracy than that of GPT-3.5 and higher than or equal to that of GPT-4. For type X questions, GPT-4 and GPT-5 scored significantly higher than GPT-3.5, whereas no significant difference was observed between GPT-4 and GPT-5. For type X2 questions, GPT-5 scored significantly higher than GPT-3.5 and GPT-4, whereas no significant difference was observed between GPT-3.5 and GPT-4. This indicates that for simple single-answer questions, the ability of GPT-4 was sufficient. By contrast, GPT-5 was required to address more complicated questions. The recent developments in GPT models are reflected clearly in these results. In contrast, for type X3 and XX questions, no significant difference was observed among the models. These questions required GPT models to determine the correct combinations of choices, which may have increased the complexity of the task. Therefore, the processing complexity required for these tasks may have limited performance differences among the models. Further developments in GPT models may enhance their performance in future. Regarding performance according to the level of required taxonomy, GPT-4 and GPT-5 showed significantly higher accuracies than GPT-3.5 on type I questions, which required recall.

Our findings clearly revealed the differences in the abilities and characteristics of the models. Although the number of parameters in a model, which is associated with knowledge capacity and corresponds to the functional capability of the human brain, remains unknown, GPT-5 is believed to have more parameters than GPT-4 and substantially more than GPT-3.5. The maximum number of tokens, which is associated with the word capacity of the responses, is also unknown. However, GPT-5 is considered to have a higher number than GPT-4, which is believed to have a higher number than GPT-3.5. It is hence convincing that GPT-5 showed higher performance than that of GPT-3.5 and GPT-4 due to their differences.

However, it is important to consider the phenomenon called hallucination, in which AI generates incorrect responses results caused by learning old or incorrect data. This tendency was observed in this study as well as in previous studies [4, 22, 24]. This weakness indicates the current limitations of generative AI, including ChatGPT. Because its learning patterns are still developing and based on English data, users should not accept its responses uncritically, especially those regarding medical or legal topics, as following incorrect information in such cases can have serious consequences. Instead, generative AI should be used as a reference tool along with other reliable sources.

In conclusion, GPT-5 exceeded the passing score when it answered questions taken from the JSOMR board-certification examination. These findings suggest that GPT-5 has achieved a level of text-based knowledge comparable to that assessed in the JSOMR board-certification examination, and it would be an effective tool for learning and preparing for the examination. However, passing a written examination does not indicate equivalence to a board-certified specialist, as clinical expertise in oral and maxillofacial radiology requires comprehensive abilities, including image interpretation, clinical decision-making, and practical experience.

### Limitations

Each question was entered into ChatGPT in Japanese with no special prompt to reflect the actual examination environment. Hence, it is possible that the accuracy would have improved if these questions had been translated into English and entered them with optimized prompts. This study evaluated only text-based knowledge and did not assess other essential abilities of oral and maxillofacial radiologists such as image interpretation, clinical decision-making, and practical experience. Although GPT models demonstrated high performance, the possibility of incorrect responses or hallucinations remains a concern.

## Data Availability

No datasets were generated or analysed during the current study.
